# Sedentary Behavior, Physical Activity, and Health of Workers in Chile According to the National Health Survey-2017

**DOI:** 10.3390/epidemiologia6010015

**Published:** 2025-03-20

**Authors:** Jaime Leppe Zamora, Marco Leppe Zamora, Sonia Roa-Alcaino, Olga Lucía Sarmiento

**Affiliations:** 1 School of Physiotherapy, Facultad de Medicina Clínica Alemana, Universidad del Desarrollo, Av. Plaza 680, Santiago 7610658, Chile; sroa@udd.cl; 2 Occupational Health and Community Supervisor, Buses Hualpén, Puerto Santiago 195, Santiago 9061267, Chile; marco.leppe@buseshualpen.cl; 3 School of Medicine, Universidad de los Andes, Cra 1 Nº 18ª-12, Bogotá 111711, Colombia; osarmien@uniandes.edu.co

**Keywords:** sedentary behavior, musculoskeletal symptoms, occupational groups, physical activity

## Abstract

Background/Objectives: Sedentary behavior (SB) and physical activity (PA) are key determinants of health in occupational settings. This study aimed to analyze the levels of SB, PA, and their associations with health outcomes among Chilean workers using data from the National Health Survey-2017. Methods: A secondary analysis of 2042 workers aged ≥18 years was conducted. Occupations were classified using ISCO-08, and SB/PA were assessed using the Global Physical Activity Questionnaire (GPAQ). Health outcomes included musculoskeletal symptoms, hypertension, diabetes mellitus, metabolic syndrome, and cardiovascular risk. Results: Of the participants, 49.8% were women, and the mean age was 45 years (±13.7). The median SB was 120 min/day, with 32.6% accumulating ≥4 h/day. “Managers” exhibited the highest SB (median: 270 min/day). The median total PA was 123 min/day, and “Skilled agricultural, forestry, and fishery workers” reported the highest PA (median: 330 min/day). The SB (≥4 h/day) was significantly associated with musculoskeletal symptoms (OR: 1.61, 95% CI: 1.21–2.14) and hypertension (OR: 1.53, 95% CI: 1.07–2.18). PA showed no significant protective effect. Conclusions: SB and PA vary significantly across occupational groups. SB is associated with musculoskeletal symptoms. Health promotion programs should be tailored to specific occupational groups.

## 1. Introduction

Sedentary behavior (SB) and physical activity (PA) are recognized risk factors for non-communicable diseases, particularly in occupational settings, as highlighted by the World Health Organization (WHO) [1,2,3]. PA is defined as any bodily movement produced by skeletal muscles that requires energy expenditure and can be classified by intensity as light, moderate, or vigorous [4]. Conversely, SB is defined as any waking behavior characterized by sitting, reclining, or lying down with an energy expenditure of ≤1.5 metabolic equivalents [5]. Modernization and technological advances in the workplace have significantly increased SB, especially among office workers and vehicle drivers [6]. The workplace is a critical domain where both behaviors coexist, and understanding their distinct health effects is essential [7]. PA has well-documented health benefits, including a reduced risk of cardiovascular diseases, metabolic disorders, and mental health conditions [2]. However, its impact on health varies depending on intensity, duration, and the domain in which it occurs [8,9].

On the other hand, prolonged SB has been linked to adverse health outcomes, including all-cause mortality, cardiovascular diseases, type 2 diabetes, metabolic syndrome, and musculoskeletal symptoms. These associations have been demonstrated in studies utilizing both self-reported data and objective measurements, such as accelerometers, although sedentary behavior is often underestimated [10,11,12,13,14] For example, in Chile, data from the Chilean National Health Survey-2017 reveal that self-reported SB averages 3 h/day, whereas accelerometer-based measurements indicate an average of 9 h/day [15,16,17]. Despite this discrepancy, subjective data have identified significant associations between SB and several health conditions, such as cardiovascular risk factors, type 2 diabetes, and cognitive impairment [18,19]. These findings underscore the critical need to address SB as a modifiable risk factor, particularly in occupational settings.

SB and PA patterns vary between working and non-working populations, with notable differences across occupational groups; for instance, office workers spend more than 65% of their working hours sitting, with nearly half of this time accumulated in prolonged bouts [20,21]. These differences underscore the need to identify high-risk occupational groups and implement tailored interventions, aligning with global efforts by the WHO and the Global Observatory for Physical Activity to reduce sedentary time and promote physical activity as strategies to improve workforce health [2,22,23].

The primary objective of this study is to determine the levels of SB and PA and their association with health outcomes in the working population, using data from the Chilean National Health Survey-2017 and the International Standard Classification of Occupations 08 (ISCO-08) classification system.

## 2. Materials and Methods

### 2.1. Study Design

A secondary analysis of the Chilean National Health Survey-2017 (CNHS-2017) was conducted. The CNHS-2017 is a nationally representative cross-sectional household survey based on a stratified, multistage probabilistic sample of n = 6233 participants. It covers all regions of Chile with both urban and rural representativeness. Detailed information on the methodological characteristics of the CNHS-2017 is available on the Ministry of Health’s website [24].

### 2.2. Participants

**Figure 1:**
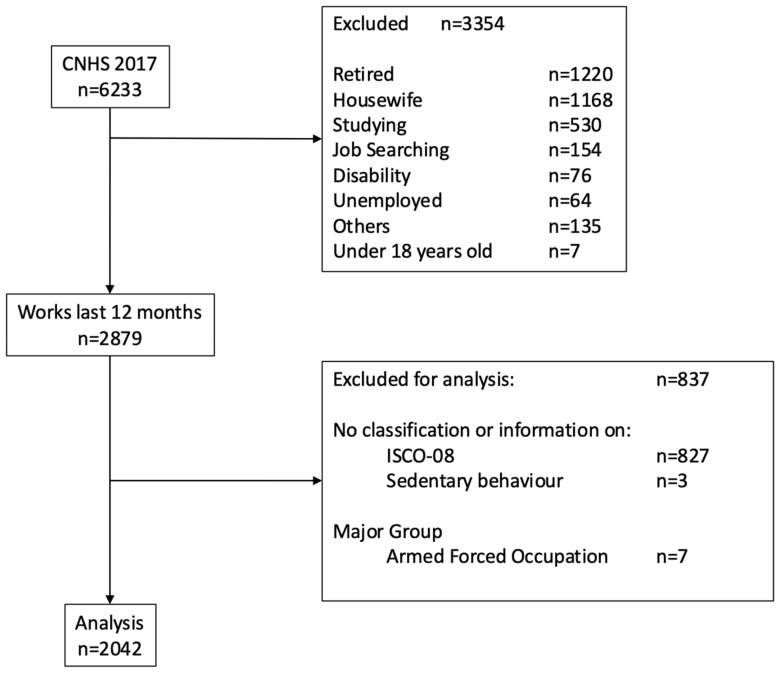
Participant flow chart.

All workers over 18 years of age who reported paid employment in the past 12 months were included. A total of n = 2042 participants were eligible for analysis. Among the excluded participants, those classified in the “Other” category (n = 135) corresponded to persons who did not answer the question on occupational classification, worked for a family member without pay, were renters, declared that they were not looking for work, or were in an extraordinary situation that they did not want to identify. Figure 1 shows the flow chart of participant analysis.

### 2.3. Classification of Occupations

Occupations were categorized using the International Standard Classification of Occupations 08 (ISCO-08) [25]. This system organizes jobs into defined groups based on required tasks and skill levels. The classification comprises ten internationally codified groups, numbered from 0 to 9. Workers were assigned according to “Major Occupational Groups” (MOGs).

The MOGs were as follows:

1. Managers
2. Professionals
3. Technicians and associate professionals
4. Clerical support workers
5. Service and sales workers
6. Skilled agricultural, forestry, and fishery workers
7. Craft and related trades workers
8. Plant and machine operators, and assemblers
9. Elementary occupations
10. Armed forces occupations

Group 0, corresponding to the armed forces, was excluded from the analysis due to the small sample size (n = 7).

### 2.4. Measurements

The PA and SB were assessed using the Global Physical Activity Questionnaire (GPAQ v2), a standardized instrument developed by the World Health Organization (WHO) to measure PA levels across diverse populations. GPAQ categorizes PA into three domains: work, travel, and leisure time. Respondents were asked about the time (minutes per day) spent engaging in moderate and vigorous-intensity PA during a typical week for each domain. The questionnaire collects:

- Frequency (days per week) of PA in each domain.
- Duration (minutes per day) of moderate and vigorous PA in each domain.

The total PA minutes per week were obtained for each domain separately and summed across all domains. PA was classified according to WHO criteria as:

- Sufficiently active (≥600 MET-min/week).
- Insufficiently active (<600 MET-min/week).

According to WHO criteria, individuals were classified as sufficiently active if they achieved at least 150 min of moderate physical activity per week, 75 min of vigorous physical activity per week, or an equivalent combination totalling at least 600 MET-minutes per week [2].

SB was assessed using a single-item GPAQ question: “How much time do you usually spend sitting or reclining on a typical day?”.

The validity and reliability of GPAQ have been supported by international validation studies, which demonstrated its comparability with accelerometer-based PA assessments. We followed the GPAQ Analysis Guide (WHO) to ensure accurate classification and interpretation of PA and SB levels [26].

Additionally, SB was analyzed as a continuous variable in hours per day and categorized into quartiles: Q1 (<1 h/day), Q2 (1–<2 h/day), Q3 (2–<4 h/day), and Q4 (≥4 h/day). SB was further dichotomized for regression analysis using a cutoff of ≥4 h/day.

The GPAQ was incorporated as a measurement tool in CNHS in 2010. Its validity and reliability were tested using accelerometers and test–retest methods, yielding results consistent with the literature: Spearman’s rho = 0.35 (*p* < 0.01) and Kappa = 0.24 (*p* < 0.01), with an agreement rate of 71.9% for PA. For SB, the GPAQ demonstrated a Spearman’s rho of 0.23 (*p* < 0.001) [15,17].

Both SB and PA time were truncated at 960 min per day, following the consensus to account for 8 h of sleep [26].

### 2.5. Health Conditions

Several health conditions were analyzed in this study due to their recognized association with SB and PA in the working population [15,17]. These conditions include musculoskeletal symptoms, hypertension, diabetes mellitus, metabolic syndrome, cardiovascular risk, and overweight. The CNHS report provides the operational definitions for each variable in detail [24].

#### 2.5.1. Musculoskeletal Symptoms (MKS)

MKSs were assessed using the CCQ-ILAR questionnaire, a validated instrument designed to evaluate non-traumatic pain. This tool identifies the affected anatomical regions and provides detailed information on the duration and intensity of symptoms [27,28].

#### 2.5.2. Hypertension (HT)

HT was defined as a measured systolic blood pressure ≥140 mmHg, diastolic blood pressure ≥90 mmHg, or self-reported antihypertensive treatment. 

#### 2.5.3. Diabetes Mellitus (DM)

DM was determined as fasting blood glucose levels >126 mg/dL.

#### 2.5.4. Metabolic Syndrome (MetS)

MetS was defined if participants met at least three of the following five criteria:

- Blood pressure ≥130/85 mmHg or treatment for hypertension.
- Triglycerides ≥150 mg/dL.
- Fasting glucose ≥100 mg/dL or treatment for diabetes.
- Waist circumference >80 cm (women) or >90 cm (men), following WHO standards.
- Low HDL cholesterol levels (<40 mg/dL for men, <50 mg/dL for women).

#### 2.5.5. Cardiovascular Risk (CVR)

CVR was assessed using the Framingham Risk Score, adapted for the Chilean population. This tool estimates the 10-year probability of cardiovascular events or strokes in individuals without prior history. Participants were categorized based on this risk level.

#### 2.5.6. Overweight

Overweight was defined according to the WHO criteria as a Body Mass Index (BMI) ≥ 25 kg/m^2^. BMI was calculated using the standard formula: weight in kilograms divided by height in meters squared (kg/m^2^). This classification encompasses individuals who are overweight (BMI 25.0–29.9 kg/m^2^) and those with obesity (BMI ≥ 30 kg/m^2^).

### 2.6. Statistical Analysis

A descriptive analysis of the study population was conducted, focusing on sociodemographic characteristics, MOGs, health conditions, and SB and PA. This included central tendency and dispersion measures for continuous variables and absolute and relative frequencies for categorical variables. The SB and PA were reported as medians and interquartile ranges across MOGs, and group comparisons were performed using the Kruskal–Wallis test.

To evaluate the association between health outcomes and PA and SB by MOGs, multiple logistic regression adjusted for sex, age, education, and residence area was performed. Odds ratios (OR) and 95% confidence intervals (95% CI) were calculated for all models to quantify the strength and significance of associations. All analyses were conducted using STATA version 15.1^®^ software.

### 2.7. Ethics Approval

This study did not require ethics approval and participant consent, as it utilized a secondary database made available by the Chilean Ministry of Health for research purposes.

## 3. Results

### 3.1. Demographic Characteristics

A total of 2042 participants were included in the study. Of these, 49.8% were women. The median age was 45 years (±13.6). Most participants (88.2%) resided in urban areas, and 57.3% had completed 8–12 years of education.

The most prevalent occupational group in the study was “Service and sales workers” (24.7%), followed by “Elementary occupations” (16.5%). Among women, the most common group was “Service and sales workers” (32.7%), while among men, it was “Craft and related trades workers” (24.5%). The second most prevalent group for women was “Elementary occupations” (22.0%), and for men, it was “Plant and machine operators, and assemblers” (16.9%). Table 1 summarizes these characteristics.

### 3.2. Health Conditions

The prevalence of health conditions in descending order was as follows: MetS: 41.5%, MSK: 38.6%, HT: 28.8%, CVR: 22.2%, and DM: 12.8%. When stratified by sex, the most prevalent conditions among women were MSK: 45.4% and MetS: 38.1%, whereas in men, MetS: 45.0% and HT: 33.4%, were the most common. The prevalence of overweight (BMI ≥ 25 kg/m^2^) in the study population was 79.1%. Statistically significant differences were observed for all health conditions between sexes, except for the CVR, DM, and overweight. The distribution of health conditions stratified by sex is detailed in Table 1.

### 3.3. Sedentary Behavior and Physical Activity

The daily accumulated SB was a median of 120 min/day, and 32.6% of the population accumulated more than 4 h of daily SB. Analyzed by sex, women spent more time in SB, with 34.6% accumulating over 4 h daily compared to 30.6% for men. Regarding PA, 71.7% of the population met the WHO criteria for sufficient PA. The total daily PA across all domains was median of 123 min/day; within the travel domain, the median was 20 min/day, while in both the work and leisure time domains, the median was 0 min/day. Men reported higher levels of physical activity than women across all domains, and this difference was statistically significant (*p* < 0.01). The detailed distribution of the total physical activity time and domain, stratified by sex, is presented in Table 1. 

### 3.4. Major Occupational Group and Patterns of SB and PA

**Figure 2:**
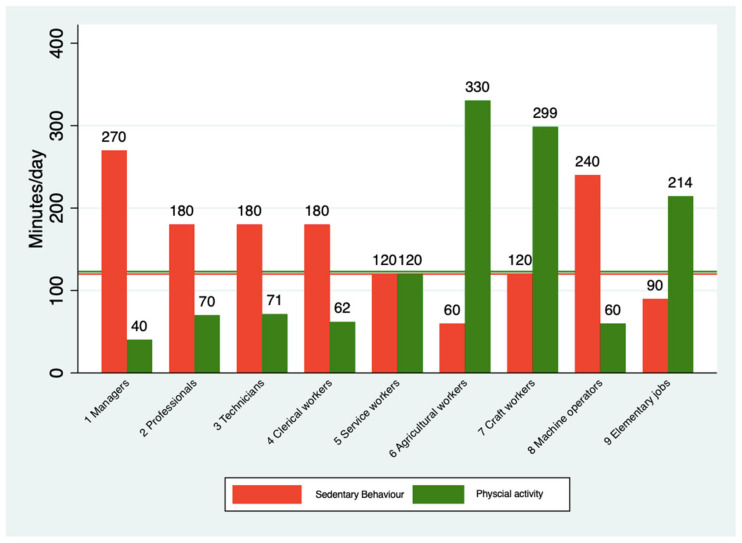
Sedentary behavior (SB) and physical activity (PA) presented as medians (minutes/day) for the major occupational groups. The horizontal lines represent all groups’ median values for SB and PA. (1) Managers, (2) Professionals, (3) Technicians and associate professionals, (4) Clerical support workers, (5) Service and sales workers, (6) Skilled agricultural, forestry, and fishery workers, (7) Craft and related trades workers, (8) Plant and machine operators, and assemblers, (9) Elementary occupations.

“Managers” reported the highest SB with a median of 270 min/day, while “Skilled agricultural, forestry, and fishery workers” showed the lowest SB with a median of 60 min/day. Regarding PA, “Skilled agricultural, forestry, and fishery workers” reported the highest levels, with a median of 330 min/day, whereas “Managers” reported the lowest PA with a median of 40 min/day. Figure 2 illustrates the total PA and SB spent according to MOGs. 

The PA levels varied significantly across domains. In the PA-Work, the median was 0 min/day across all groups, and for “Craft and related trades workers”, the median was 206 min/day, reporting the highest PA. In the PA-Travel, the median was 20 min/day across all groups, “Skilled agricultural, forestry, and fishery workers” reported the highest time with a median of 44 min/day. The PA-Leisure reported a median of 0 min/day across groups. A detailed of the SB and PA patterns according to MOGs is provided in Table 2. 

### 3.5. Association Between Health Outcomes and, SB and PA

Prolonged sedentary behavior (SB ≥ 4 h/day) demonstrated significant associations with specific health outcomes. Participants accumulating ≥4 h/day of SB exhibited higher odds of MSK (OR: 1.61, 95% CI: 1.21–2.14) and HT (OR: 1.53, 95% CI: 1.07–2.18) after adjustment for MOGs, sex, age, education, and residence area. The association between SB and health outcomes varied across occupational groups. Among “Skilled agricultural, forestry, and fishery workers” and “Craft and related trades workers”, the odds of MSK were particularly elevated (OR: 2.30, 95% CI: 1.10–4.82; and OR: 2.21, 95% CI: 1.23–3.96, respectively).

Sex and age also influenced the associations observed. Women were more likely to report MSK (OR: 2.05, 95% CI: 1.67–2.51), whereas men exhibited a higher likelihood of HT. Age remained a consistent risk factor across all health outcomes. Detailed estimates of odds ratios and confidence intervals are provided in Table 3.

To further explore the role of age in these associations, we performed an additional analysis by stratifying the sample into two age groups: <40 years and ≥40 years. The results (view Table S1) show that the association between prolonged sedentary behavior (≥4 h/day) and musculoskeletal symptoms remains significant in both strata. The association with hypertension was significant only in the over-40 age stratum.

No significant associations were identified between adherence to the WHO PA criteria and the evaluated health outcomes in either crude or adjusted models (Table S2).

## 4. Discussion

This study used nationally representative data to investigate the relationship between SB, PA, and health outcomes among workers. Significant differences in SB and PA levels were identified across occupational groups. Sedentary occupations accumulate more SB time than manual occupations, which, although characterized by higher levels of PA, do not show protective health benefits. Specifically, SB (≥4 h/day) was associated with adverse health outcomes such as MKS (adjusted OR = 1.61; 95% CI: 1.21–2.14) and HT (adjusted OR = 1.53; 95% CI: 1.07–2.18). This association remained in an age-stratified analysis but only for musculoskeletal symptoms, while the association with hypertension was significant only in participants aged 40 years and older.

### 4.1. Comparison with Previous Studies

Our findings align with studies such as Sung et al. (2021), which emphasize the associations between occupations and health behaviors [21]. Consistent with their results, we observed that sedentary occupations exhibited higher levels of SB, with managers reporting a median of 270 min per day. In contrast, manual occupations, such as agricultural workers, demonstrated significantly higher levels of total PA, with a median of 330 min/day. However, our definition of “high SB” (≥4 h/day) differs from the 7 h/day threshold proposed by Sung. This methodological choice reflects the limitations of the self-reported SB data from the Chilean National Health Survey. Aguilar and Leppe (2016) [15] found that self-reported measures, such as the single-item question from the GPAQ, tend to substantially underestimate the actual SB compared to objective methods such as using an accelerometer. This underestimation, averaging nearly 5 h per day, informed our decision to use a lower cutoff, addressing the systematic bias in self-reported measures and enhancing the validity of our findings [15].

Studies such as Saidj et al. (2015) have reported that occupations with high workplace SB also exhibit elevated sedentary time outside working hours [29]. While our study did not directly evaluate this aspect, the low reported time spent on leisure-time PA, 13 min/day on average, was considerably lower than the 30 min/day observed in developed countries, as reported by Kitano et al. (2022), which may indicate a sustained SB pattern [30].

Shivakumar et al. (2023) observed a high risk of musculoskeletal disorders due to repetitive postures and excessive physical effort in manual occupations [31]. This is consistent with our observation of significant associations between MKS and occupational groups such as agricultural workers and machine operators (OR > 2.0). Additionally, studies such as that by Parry (2019) have explored interventions to reduce SB in office workers, which could apply to similar contexts [32].

### 4.2. The Physical Activity Paradox

A relevant finding of this study is the physical activity paradox, originally described by Holtermann et al. (2012) [33]. This paradox suggests that while leisure-time physical activity provides substantial health benefits, physical activity performed at work may lead to negative health outcomes due to the nature and conditions under which it occurs. Our findings reinforce this paradox, as workers in manual occupations, despite reporting high levels of PA, did not exhibit significant protective health effects.

Recent evidence provides further insights into the mechanisms behind this paradox. Studies indicate that PA at work is often low intensity but prolonged, lacking adequate recovery periods, and frequently involves repetitive tasks and biomechanical strain. These conditions can contribute to sustained cardiovascular stress, increased blood pressure, and chronic inflammation, which may explain the absence of expected health benefits [8,34]. For example, Gupta et al. (2020) reported that moderate-to-vigorous PA reduces the risk of prolonged sickness absence by 20% when performed during leisure time but increases it by 15% when performed at work, highlighting the importance of differentiating PA by context rather than volume alone [35].

Given these findings, future interventions should focus not on reducing PA at work but on redesigning work environments to mitigate its adverse effects. Strategies such as task variation, structured active breaks, ergonomic modifications, and complementary leisure-time PA could help improve worker health outcomes while maintaining productivity [34].

### 4.3. Practical and Theoretical Relevance

This study expands the understanding of the impact of the occupational context on SB and PA in high-income but developing countries like Chile, providing a comparison with studies conducted in developed countries [20,21,29]. The findings underscore the importance of developing tailored interventions to reduce SB and increase leisure-time PA. Additionally, as de Govaerts et al. (2021) highlighted, implementing ergonomic strategies to prevent musculoskeletal disorders among manual workers is crucial [36].

### 4.4. Strengths and Limitations

The limitations include the cross-sectional design, which prevents causal inferences, and the use of self-reported data, which are susceptible to recall bias or social desirability bias. These limitations could have been mitigated using objective measurements such as accelerometers to assess SB and PA. Moreover, future studies could employ longitudinal designs to explore causal relationships more precisely.

Additionally, residual and unmeasured confounding must be considered. While we adjusted for key sociodemographic and occupational factors, other potential confounders, such as dietary habits, genetic predisposition, and work-related psychosocial stress, were not accounted for. These factors could influence the observed associations and should be considered in future research.

However, the strengths include using nationally representative data and standardized occupational classification (ISCO-08), enabling international comparisons, and identifying groups for targeted interventions.

### 4.5. Future Implications

The results from this study, alongside existing scientific evidence, reinforce the concept that reducing SB and increasing PA during non-work hours could be an effective strategy for improving workers’ health. Our findings and the broader evidence base highlight the need for health promotion interventions—grounded in policy, education, or environmental changes—that are differentiated by occupational groups. Initiatives could begin by reducing SB, as advocated by Dogra et al. (2022), and then progress to increasing PA levels [37]. Specific interventions, such as standing desks, computer prompts, and active workstations, should be prioritized for office workers [38,39,40]. Additionally, future research should prioritize longitudinal studies to establish causal relationships and assess the effectiveness of intervention strategies across different occupational and cultural contexts. Given the inherent limitations of cross-sectional designs, randomized controlled trials, Mendelian randomization approaches, and causal mediation analysis can provide stronger causal inferences regarding the associations between sedentary behavior, physical activity, and health outcomes. Mendelian randomization offers a method to infer causality by using genetic variants as instrumental variables, reducing the risk of residual confounding. Causal mediation models can further elucidate the underlying pathways linking SB and PA to health outcomes, distinguishing direct and indirect effects. Furthermore, cohort studies with repeated measurements are essential for refining these associations over time [38].

Intervention strategies for workplace physical activity must consider occupational differences. Studies show that white-collar workers engage in lower workplace activity but may compensate in leisure time, while blue-collar workers have physically demanding jobs but lower structured exercise habits [41]. Programs for office workers should focus on reducing sedentary behavior [42], while additional research is needed to determine the most effective interventions for manual laborers.

## 5. Conclusions

This study highlights the significant variability in SB and PA levels across occupational groups among Chilean workers. Sedentary occupations, such as managers, professionals, and clerical support workers, were associated with adverse health outcomes, including musculoskeletal symptoms and hypertension. In contrast, physically demanding jobs, such as those in agricultural and craft sectors, exhibited high levels of occupational PA, but no significant associations were observed between PA and protective health effects, which is consistent with the physical activity paradox.

These findings underscore the importance of workplace health strategies that prioritize reducing SB and promoting leisure-time PA to improve overall health and well-being among workers. Tailored interventions should address the specific risks associated with occupational demands while encouraging recovery and leisure-time PA to maximize health benefits.

## Figures and Tables

**Table 1:** Sociodemographic characteristics, type of occupation, health condition, physical activity, and sedentary behavior of the study population, according to the Chilean National Health Survey-2017 by sex.

	Total	Female	Male	*p*-Value
n (%)	2042 (100)	1017 (49.8)	1025 (50.2)	
Age (years)	45 (33–56)	44 (33–54)	46 (33–57)	0.015
Age group				
18–24 years	139 (6.8)	64 (6.3)	75 (7.3)	0.059
25–44 years	880 (43.1)	466 (45.8)	414 (40.4)	
45–64 years	891 (43.6)	430 (42.3)	461 (45.0)	
≥64 years	132 (6.5)	57 (5.6)	75 (7.3)	
Residence area				
Urban	1802 (88.2)	894 (87.9)	908 (88.6)	0.63
Education				
Low (<8 years)	252 (12.4)	121 (12.0)	131 (12.9)	0.049
Medium (8–12 years)	1161 (57.3)	557 (55.2)	604 (59.3)	
High (≥13 years)	614 (30.3)	331 (32.8)	283 (27.8)	
Major Occupational Group				
1. Managers	74 (3.6)	39 (3.8)	35 (3.4)	<0.001
2. Professionals	243 (11.9)	151 (14.8)	92 (9.0)	
3. Technicians and associate professionals	214 (10.5)	102 (10.0)	112 (10.9)	
4. Clerical support workers	97 (4.8)	63 (6.2)	34 (3.3)	
5. Service and sales workers	504 (24.7)	333(32.7)	171 (16.7)	
6. Skilled agricultural, forestry, and fishery workers	64 (3.1)	20.(2.0)	44 (4.3)	
7. Craft and related trades workers	309 (15.1)	58 (5.7)	251 (24.5)	
8. Plant and machine operators, and assemblers	200 (9.8)	27 (2.7)	173 (16.9)	
9. Elementary occupations	337 (16.5)	224 (22.0)	113 (11.0)	
Health conditions				
Overweight	1416 (79.1)	703 (77.9)	713 (80.3)	0.22
Metabolic syndrome	474 (41.5)	217 (38.1)	257 (45.0)	0.017
Musculoskeletal symptoms	788 (38.6)	462 (45.4)	326 (31.8)	<0.001
Hypertension	527 (28.8)	223 (24.2)	304 (33.4)	<0.001
Cardiovascular risk	253 (22.2)	131 (22.9)	122 (21.4)	0.028
Diabetes	220 (12.8)	110 (12.7)	110 (12.9)	0.88
Sedentary behavior P_50_ (P_25_–P_75_)				
Total (min/day)	120 (60, 243)	120 (60–240)	120 (60–300)	0.008
Sedentary behavior (quartiles)				
<1 h	364 (17.8)	201 (19.8)	163 (15.9)	0.065
1–2 h	403 (19.7)	206 (20.3)	197 (19.2)	
2–4 h	609 (29.8)	299 (29.4)	310 (30.2)	
>4 h	666 (32.6)	311 (30.6)	355 (34.6)	
Physical activity P_50_ (P_25_–P_75_)				
Total (min/day)	123 (23–381)	86 (15–320)	180 (34–443)	<0.001
Work (min/day)	0 (0–257)	0 (0–214)	23 (0–334)	<0.001
Travel (min/day)	20 (0–60)	17 (0–51)	26 (0–86)	<0.001
Leisure (min/day)	0 (0, 13)	0 (0–0)	0 (0–19)	<0.001
Sufficiently active (WHO criteria)	1408 (71.7)	647 (66.4)	761 (77.0)	<0.001

Data presented as median (P25–P75), absolute frequency (relative frequency) as appropriate. Major occupational groups according to the International Standard Classification of Occupations (ISCO-08).

**Table 2:** Sedentary behavior and physical activity of Chilean workers using the International Standard Classification of Occupations (ISCO-08), according to the Chilean National Health Survey-2017.

Major Occupational Groups	SB-Total (min/day)	SB ≥ 4 (hours)	PA-Total (min/day)	PA-Travel (min/day)	PA-Work (min/day)	PA-Leisure (min/day)	Sufficiently Active
Total	120 (60–243)	32.6%	123 (223–381)	20 (0–60)	0 (0–257)	0 (0–13)	71.7%
1. Managers	270 (120–480)	59.5%	40 (0–158)	0 (0–37)	0 (0–21)	0 (0–17)	56.9%
2. Professionals	180 (60–360)	46.9%	70 (17–250)	17 (0–60)	0 (0–86)	0 (0–26)	67.5%
3. Technicians and associate professionals	180 (90–363)	47.2%	71 (17–266)	18 (0–60)	0 (0–86)	0 (0–26)	66.0%
4. Clerical support workers	180 (60–420)	47.4%	62 (11–300)	15 (0–60)	0 (0–129)	0 (0–17)	57.6%
5. Service and sales workers	120 (60–240)	27.6%	120 (26–352)	21 (0–79)	0 (0–214)	0 (0–0)	70.4%
6. Skilled agricultural, forestry, and fishery workers	60 (30–180)	15.6%	330 (194–588)	44 (4–240)	189 (0–420)	0 (0–0)	87.3%
7. Craft and related trades workers	120 (60–180)	20.4%	299 (66–489)	25 (0–60)	206 (0–386)	0 (0–17)	86.3%
8. Plant and machine operators, and assemblers	240 (60–480)	53.5%	60 (0–408)	0 (0–41)	0 (0–310)	0 (0–9)	56.8%
9. Elementary occupations	90 (47–180)	12.5%	214 (39–424)	25 (0–86)	69 (0–309)	0 (0–0)	80.1%

SB: Sedentary behavior. PA: Physical activity. Sufficiently active according to WHO criteria. Data presented in median (P_25_—P_75_) and percentage %. (min/day) minutes per day.

**Table 3:** Association between health outcomes and sedentary behavior in the Chilean National Health Survey-2017.

Unadjusted Model (95%CI)					
Sedentary Behavior (Quartile)	Musculoskeletal Symptoms	Hypertension	Diabetes	Metabolic Syndrome	Cardiovascular Risk
<1 h (reference)	reference	reference	reference	reference	reference
1–2 h	**1.34 (1.01–1.80)**	**1.44 (1.04–2.00)**	0.92 (0.59–1.44)	1.21 (0.83–1.78)	1.15 (0.74–1.80)
2–4 h	1.26 (0.96–1.65)	1.10 (0.81–1.49)	0.73 (0.48–1.11)	0.90 (0.63–1.28)	0.90 (0.59–1.37)
>4 h	1.28 (0.98–1.67)	1.03 (0.76–1.39)	0.89 (0.60–1.34)	1.01 (0.71–1.43)	0.91 (0.60–1.38)
**Adjusted Model OR (95%CI)**					
**Sedentary Behavior** **(Quartile)**	**Musculoskeletal Symptoms**	**Hypertension**	**Diabetes**	**Metabolic Syndrome**	**Cardiovascular Risk**
<1 h	reference	reference	reference	reference	reference
1–2 h	**1.43 (1.06–1.94)**	**1.67 (1.15–2.42**)	0.98 (0.61–1.55)	1.27 (0.84–1.90)	1.35 (0.83–2.21)
2–4 h	**1.37 (1.04–1.82)**	1.31 (0.92–1.85)	0.79 (0.51–1.22)	0.97 (0.67–1.41)	1.04 (0.66–1.66)
>4 h	**1.60 (1.20–2.13)**	**1.52 (1.06–2.19)**	1.03 (0.66–1.60)	1.19 (0.81–1.74)	1.17 (0.73–1.87)
Major Occupational Groups					
1. Managers	reference	reference	reference	reference	reference
2. Professionals	1.37 (0.76–2.47)	0.97 (0.45–2.08)	1.40 (0.49–4.06)	0.97 (0.46–2.06)	0.80 (0.31–2.08)
3. Technicians	1.61 (0.89–2.93)	0.90 (0.42–1.93)	1.84 (0.65–5.19)	0.84 (0.39–1.78)	0.73 (0.28–1.87)
4. Clerical support workers	1.76 (0.90–3.47)	1.24 (0.52–2.98)	1.36 (0.41–4.48)	2.11 (0.89–5.02)	1.19 (0.40–3.59)
5. Service and sales workers	1.65 (0.93–2.92)	1.45 (0.71–2.98)	1.19 (0.43–3.28)	1.17 (0.58–2.34)	0.85 (0.35–2.07)
6. Agricultural workers	2.26 (1.03–4.95)	0.99 (0.37–2.66)	1.57 (0.42–5.81)	0.60 (0.23–1.57)	0.60 (0.17–2.15)
7. Craft and related trades	2.01 (1.11–3.67)	1.09 (0.52–2.30)	0.82 (0.28–2.39)	1.28 (0.62–2.63)	0.48 (0.19–1.23)
8. Machine operators	2.00 (1.08–3.72)	1.26 (0.59–2.71)	1.77 (0.62–5.11)	1.56 (0.73–3.33)	1.03 (0.39–2.71)
9. Elementary occupations	1.87 (1.03–3.41)	1.49 (0.71–3.15)	1.26 (0.44–3.59)	1.09 (0.53–2.26)	0.77 (0.30–1.97)

Bold values indicate statistically significant results.

## Data Availability

Data are available on the epidemiology page of the Chilean Ministry of Health.

## Supplementary Materials

The following supporting information can be downloaded at: https://www.mdpi.com/article/10.3390/epidemiologia6010015/s1, Table S1: Association between Health Outcomes and Sedentary Behaviour in the Chilean National Health Survey 2017, Stratified by Age. Table S2: Association between Health Outcomes and Physical Activity in the Chilean National Health Survey 2017.

## Abbreviations

The following abbreviations are used in this manuscript:

SB	Sedentary behavior
PA	Physical activity
CNHS-2017	Chilean National Health Survey-2017
ISCO-08	International Standard Occupation Classification 08
GPAQ	Global Physical Activity Questionnaire
WHO	World Health Organization
OR	Odds ratio
CI	Confidence interval
MOGs	Major occupational group
MSK	Musculoskeletal symptoms
HT	Hypertension
DM	Diabetes mellitus
MetS	Metabolic syndrome
