# Supplementary material for: Sedentary Behavior, Physical Activity, and Health of Workers in Chile According to the National Health Survey-2017
Source: Epidemiologia (Basel). 2025 Mar 20;6(1):15. doi: 10.3390/epidemiologia6010015 (PMC11941120; doi:10.3390/epidemiologia6010015)
Supplement: Supplementary file 1 [file epidemiologia-06-00015-s001.zip › epidemiologia-3472641-supplementary.pdf]

Supplementary Materials

Sedentary Behavior, Physical Activity, and Health of Workers in Chile According to the National Health Survey-2017

Jaime Leppe Zamora [1,*], Marco Leppe Zamora [2], Sonia Roa-Alcaino [1] and Olga Lucía Sarmiento [3]

[1] School of Physiotherapy, Facultad de Medicina Clínica Alemana, Universidad del Desarrollo, Santiago, Chile; sroa@udd.cl
[2] Occupational Health and Community Supervisor, Buses Hualpén Santiago, Chile; marco.leppe@buseshualpen.cl
[3] School of Medicine, Universidad de los Andes, Bogotá, Colombia; osarmien@uniandes.edu.co
* Correspondence: jleppe@udd.cl

Supplementary Table S1. Association between Health Outcomes and Sedentary Behaviour in the Chilean National Health Survey 2017, Stratified by Age.

Health Outcomes	Sedentary Behaviour (Quartile)	Total OR (95% CI)	Age < 40 years OR (95% CI)	Age ≥ 40 years OR (95% CI)
Musculoskeletal Symptoms	<1 hour	ref	ref	ref
	1-2 hours	**1.43 (1.06–1.94)**	1.72 (0.97–3.06)	1.35 (0.94–1.94)
	2-4 hours	**1.37 (1.04–1.82)**	1.68 (0.99–2.85)	1.29 (0.92–1.81)
	>4 hours	**1.60 (1.20–2.13)**	**1.93 (1.15–3.23)**	**1.51 (1.06–2.16)**
Hypertension	<1 hour	ref	ref	ref
	1-2 hours	**1.67 (1.15–2.42)**	1.96 (0.54–7.12)	**1.70 (1.15–2.51)**
	2-4 hours	1.31 (0.92–1.85)	2.31 (0.70–7.57)	1.26 (0.88–1.82)
	>4 hours	**1.52 (1.06–2.19)**	2.41 (0.75–7.75)	**1.49 (1.02–2.19)**
Diabetes	<1 hour	ref	ref	ref
	1-2 hours	0.98 (0.61–1.55)	0.62 (0.13–3.03)	1.01 (0.62–1.64)
	2-4 hours	0.79 (0.51–1.22)	1.18 (0.33–4.18)	0.75 (0.46–1.20)
	>4 hours	1.03 (0.66–1.60)	1.10 (0.33–3.66)	1.07 (0.66–1.73)
Metabolic Syndrome	<1 hour	ref	ref	ref
	1-2 hours	1.27 (0.84–1.90)	1.44 (0.89–2.33)	1.46 (0.91–2.35)
	2-4 hours	0.97 (0.67–1.41)	1.01 (0.65–1.57)	1.01 (0.65–1.56)
	>4 hours	1.19 (0.81–1.74)	1.37 (0.86–2.18)	1.33 (0.84–2.10)
Cardiovascular Risk	<1 hour	ref	ref	ref
	1-2 hours	1.35 (0.83–2.21)	1.30 (0.77–2.21)	1.34 (0.79–2.28)
	2-4 hours	1.04 (0.66–1.66)	1.09 (0.66–1.80)	1.07 (0.65–1.76)
	>4 hours	1.17 (0.73–1.87)	1.17 (0.69–1.95)	1.14 (0.68–1.91)

Bold values indicate statistically significant results.

Supplementary Table S2. Association between Health Outcomes and Physical Activity in the Chilean National Health Survey 2017.

**Unadjusted Model (95%CI)**					
Physical Activity (WHO Criteria)	**Musculoskeletal Symptoms**	**Hypertension**	**Diabetes**	**Metabolic Syndrome**	**Cardiovascular Risk**
Insufficiently Active	reference	reference	reference	reference	reference
Sufficiently Active	1.07 (0.88–1.32)	1.07 (0.85–1.34)	0.76 (0.56–1.04)	0.93 (0.71–1.22)	0.83 (0.60–1.13)
**Adjusted Model OR (95%CI)**					
**Physical Activity (WHO Criteria)**	**Musculoskeletal Symptoms**	**Hypertension**	**Diabetes**	**Metabolic Syndrome**	**Cardiovascular risk**
Insufficiently Active	reference	reference	reference	reference	reference
Sufficiently Active	1.14 (0.92–1.41)	0.95 (0.72–1.25)	0.74 (0.52–1.03)	0.87 (0.64–1.17)	0.80 (0.56–1.15)
Major Occupational Groups					
1 Managers	reference	reference	reference	reference	reference
2 Professionals	1.25 (0.69–2.27)	0.94 (0.43–2.03)	1.68 (0.53–5.32)	0.96 (0.45–2.05)	0.79 (0.30–2.06)
3 Technicians	1.45 (0.80–2.65)	0.83 (0.39–1.79)	2.19 (0.71–6.80)	0.78 (0.36–1.66)	0.72 (0.28–1.84)
4 Clerical support workers	1.75 (0.88–3.47)	1.26 (0.52–3.05)	1.48 (0.40–5.40)	1.98 (0.81–4.83)	1.23 (0.41–3.71)
5 Service and sales workers	1.37 (0.77–2.44)	1.30 (0.63–2.68)	1.39 (0.46–4.22)	0.93 (0.45–1.93)	0.80 (0.33–1.97)
6 Agricultural workers	2.01 (0.91–4.43)	0.88 (0.33–2.39)	2.12 (0.53–8.48)	0.54 (0.19–1.51)	0.60 (0.16–2.16)
7 Craft and related trades	1.68 (0.91–3.08)	0.94 (0.44–2.00)	1.10 (0.35–3.48)	1.10 (0.52–2.36)	0.48 (0.19–1.25)
8 Machine operators	2.01 (1.07–3.77)	1.10 (0.51–2.38)	2.01 (0.63–6.38)	1.32 (0.59–2.94)	1.00 (0.38–2.64)
9 Elementary occupations	1.63 (0.89–2.98)	1.40 (0.66–2.97)	1.62 (0.52–5.04)	0.91 (0.42–1.96)	0.83 (0.33–2.12)
